# Penetration of inferior vena cava (IVC) filter into abdominal aorta and repairing by Dacron patch arthroplasty: A case report

**DOI:** 10.1016/j.radcr.2025.06.104

**Published:** 2025-07-26

**Authors:** Javad Jalili, Sarah Vaseghi, Mahdiyeh Baastani Khajeh, Arash Haji Kamanj Olia

**Affiliations:** a Department of Radiology, Tabriz University of Medical Sciences, Tabriz, Iran; b Department of Surgery, Tabriz University of Medical Sciences, Tabriz, Iran; c Student research committee, Tabriz University of Medical Sciences, Tabriz, Iran

**Keywords:** Thrombosis, IVC Filter, Anticoagulation, Filter migration, Vascular surgery

## Abstract

Inferior vena cava (IVC) filters are ordinarily used to prevent pulmonary embolism (PE) in patients with contraindications to anticoagulation or those with an experience of bleeding after anticoagulation therapy. We present a 54-year-old female with a complex medical history, including diabetes mellitus, who developed acute deep vein thrombosis (DVT) after right common femoral vein (RCFV) catheter insertion, necessitating intravenous Heparin therapy. Anticoagulation was discontinued due to gastrointestinal bleeding, prompting inferior vena cava (IVC) filter insertion. Prolonged dwell time led to filter penetration into the abdominal aorta with partial thrombosis, identified on imaging before planned retrieval. The patient underwent open surgery with filter removal and aortic repair using a Dacron patch. This case emphasizes the role of regular follow-up imaging and timely intervention in preventing life-threatening complications of filter retention.

## Introduction

DVT predisposes patients to PE and an increased rate of mortality. Retrievable IVC filters are widely used as a preventive measure against PE in patients with contraindications to anticoagulation or those who have experienced complications from it [1,2].

While IVC filters are generally safe and effective, they are not without potential complications, especially when left in place for extended periods [1]. These complications include filter migration, penetration of the filter struts into adjacent vascular structures (such as the Aorta), and thrombosis of the IVC or its branches [3,4]. Outcomes following IVC filter placement largely depend on the underlying condition of the patient, the presence of complications, and the promptness of any necessary interventions [5,6].

This case report describes a 54-year-old woman with multiple comorbidities who presented with acute pancreatitis due to hypertriglyceridemia and subsequently developed a DVT following RCFV catheterization for plasmapheresis. Due to gastrointestinal bleeding, anticoagulation was discontinued and an IVC filter was placed.

On delayed follow-up, imaging revealed filter strut penetration into the abdominal aorta with associated thrombosis. This report highlights the challenges associated with managing acute pancreatitis in a patient with multiple preexisting conditions and the risks linked to the use of IVC filters, emphasizing the need for careful monitoring and timely intervention.

## Case presentation

A 54-year-old female patient with a history of DM type II, cardiac arrhythmia, asthma, hypertension, and pituitary gland micro-adenoma, was referred to our center due to a 10-day history of severe epigastric pain radiating to her back.

On physical examination, she exhibited mild tenderness of the epigastric region. The bowel sounds were auscultated normally. The electrocardiogram revealed a normal sinus rhythm without any pathologic findings in the ST-T segment.

Laboratory tests reported blood sugar of 387 mg/dL (reference range up to 200 mg/dL), elevated serum lipase level of >2000 U/L (reference range, up to 160 U/L), elevated serum amylase level of >1000 U/L (reference range, up to 100 U/L), and an elevated serum triglyceride level of >1000 mg/dL (reference range, <150 mg/ dl).

Therefore, a contrast-enhanced CT scan of the abdominal and pelvic cavities was performed, which revealed a diffuse enlargement of the pancreas, along with the acute fluid collection surrounding the head, body, and tail. Additionally, a small, hypo-dense area was observed in the head of the pancreas, indicating necrosis, and confirming the diagnosis of acute necrotizing pancreatitis in association with hypertriglyceridemia. So, the patient became a candidate to receive plasmapheresis. For this purpose, a 12 French (Fr) Arrow-Clark VectorFlow double-lumen hemodialysis catheter was inserted via the RCFV on the day of admission.

Four days after the insertion of the venous catheter, the patient developed sudden swelling in her right lower extremity. Therefore, a Doppler ultrasound was performed, which reported a thrombosis in the RCFV and right external iliac vein (REIV), suggesting acute thrombosis.

**Fig. 1:**
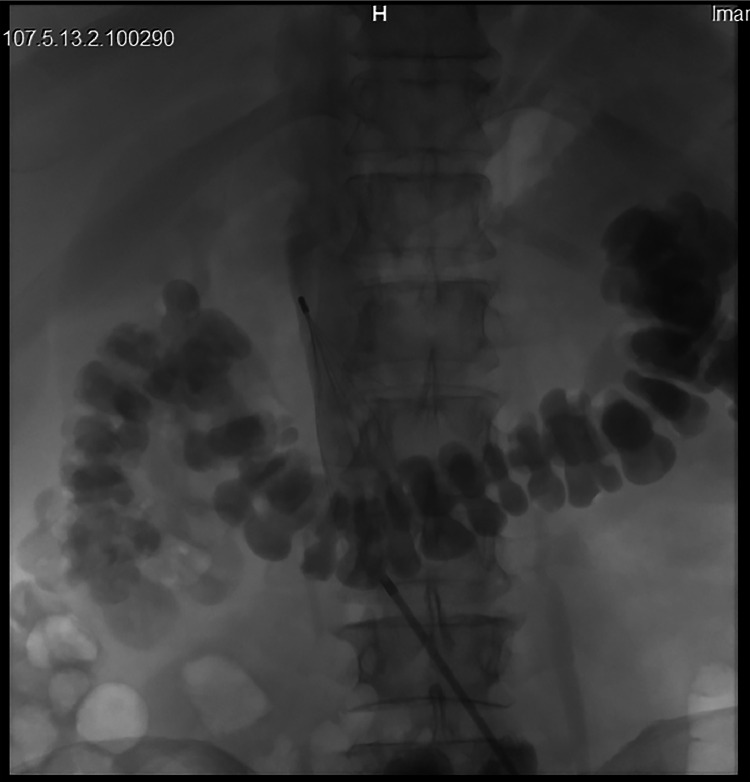
Venography image demonstrating an IVC filter placement (blue arrow) to prevent pulmonary embolism after the development of DVT following the insertion of an RCFV catheter.

Due to the acute state of the thrombosis, catheter manipulation was not recommended as it carried a high risk of embolism. Instead, a full dose regimen of IV heparin injection was started. But, 2 days later, the patient experienced GI bleeding and Heparin infusion was discontinued. The bleeding was successfully controlled by the gastroenterology service through endoscopic procedures. Therefore, the patient received an Interventional radiology consult for evaluation regarding the placement of an IVC filter, which assessed the patient's suitability for filter insertion. On the 15th day after the insertion of the catheter and the 11th day after DVT development, the Interventional Radiology team inserted a retrievable ALN Vena Cava Filter in the IVC (Fig. 1).

On the 6th day after IVC filter placement, the LCFV catheter was removed. On the 11th day after placement, the patient was discharged with strict instructions to return a week later for an evaluation of the filter and to decide on its removal. However, due to personal issues and the long travel distance, the patient returned for follow-up 37 days later (48 days after filter insertion).

**Fig. 2:**
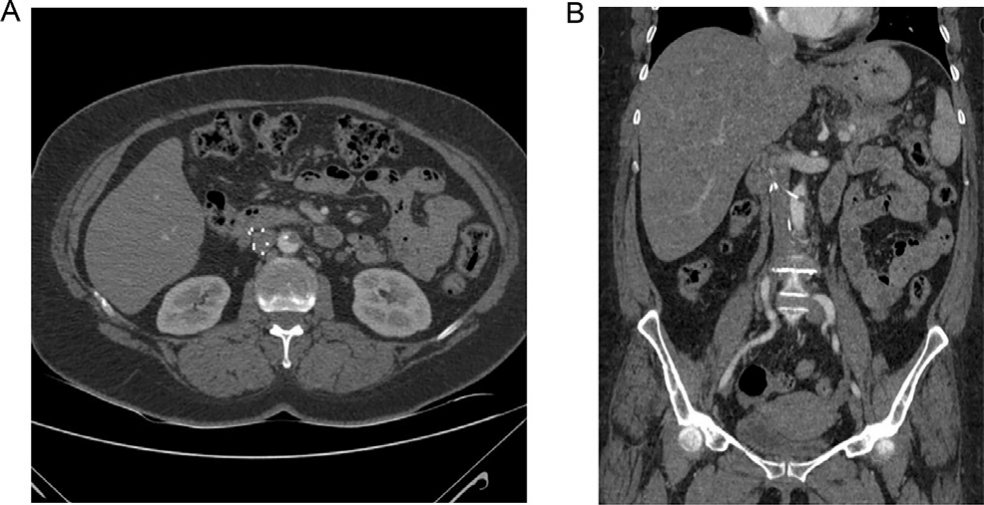
(A) Axial contrast-enhanced CT scan of the abdominal and pelvic cavities, which reported that at least three struts of the IVC filter were out of the IVC (blue arrow) and one strut was penetrated into the abdominal Aorta (red arrow). A small crescent-shaped hyperdense area is visible at the point of contact, consistent with partial aortic thrombosis. (B) Coronal contrast-enhanced CT scan demonstrating the position of the IVC filter (red arrow) and the proximity of one strut to the aortic wall. A focal crescentic hyper density at the tip of the strut (orange arrow) is consistent with partial aortic thrombosis. (yellow arrow).

Due to the prolonged duration that the filter had remained in situ, a contrast-enhanced CT scan of the abdominal and pelvic cavities was conducted 49 days after the IVC filter placement, which reported that at least three struts of the IVC filter were out of the IVC. Additionally, one strut was penetrated into the abdominal Aorta, and small partial thrombosis was seen on its tip (Fig. 2).

Therefore, based on the CT scan findings and through a multidisciplinary decision, the patient became a candidate for open surgery to remove the IVC filter that had penetrated into the Aorta.

The patient underwent consultations with the Endocrinology service for blood sugar management and the Cardiology service to evaluate the risk of general anesthesia, regarding her underlying diseases.

Informed consent and legal medical consultation were obtained regarding the potential risks of general anesthesia and the high risk of bleeding and mortality. 58 days after the placement of the IVC filter, the patient underwent open surgery by the Vascular Surgery service under general anesthesia. A midline incision was made in the patient's abdomen to access the affected area. Due to filter strut penetration into the aortic wall, both the infrarenal aorta and the IVC were opened. The thrombus and the IVC filter were carefully extracted. The aortic wall was repaired using a Dacron patch, a synthetic polyester vascular graft.

**Fig. 3:**
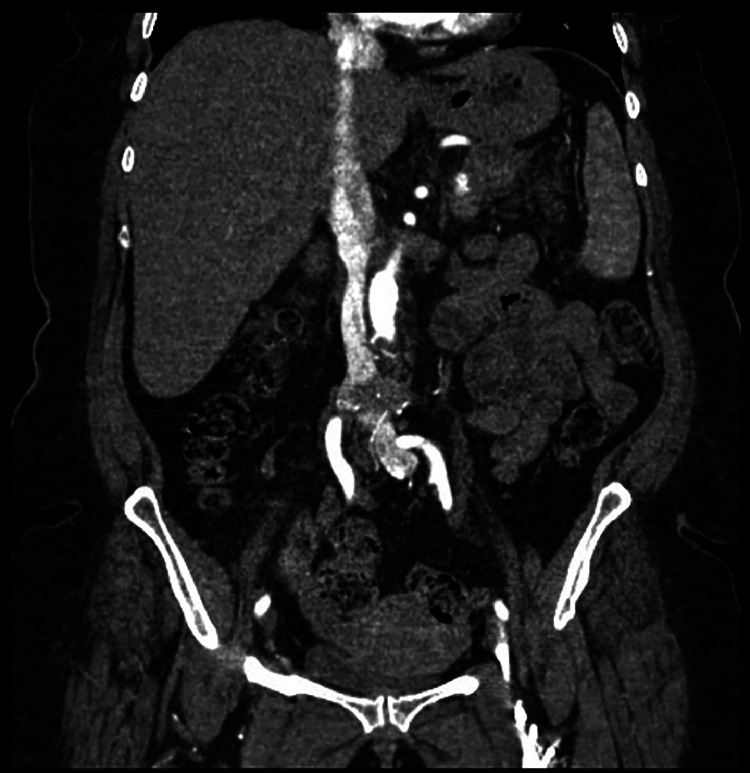
A coronal CT image obtained after open surgical removal of the IVC filter. The aorta appears intact with no visible filter struts or wall abnormalities. No residual thrombosis is observed at the prior site of strut penetration, indicating a successful repair (red arrow).

A follow-up contrast-enhanced CT scan performed after the surgical removal of the IVC filter confirmed the integrity of the aortic wall, with no residual filter struts or thrombus identified at the prior site of penetration (Fig. 3).

Following the procedure, the patient made a complete recovery and was discharged from the hospital after being admitted for 15 days. During the 1-year follow-up after the surgery, the patient did not encounter any new issues.

## Discussion

This case underscores the complexity of managing patients with multiple comorbidities who develop acute complications requiring interventional procedures, such as the insertion of an IVC filter. The patient's clinical course was complicated by the development of DVT after the insertion of an RCFV catheter for plasmapheresis, a scenario that highlights the risks associated with central venous catheterization in vulnerable patients.

Our patient’s intricate medical history places her at a heightened risk for thromboembolic complications. The development of DVT after catheterization indicates that even in the surgical setting, patients with multiple comorbidities can present significant challenges, necessitating tailored and vigilant medical management [7]. The choice to employ plasmapheresis was justified given the severe pancreatitis linked to hypertriglyceridemia, which highlights the necessity of balancing immediate therapeutic interventions against potential long-term risks.

This case underscores the critical need for comprehensive treatment protocols when managing patients at risk of thromboembolic complications, particularly those with significant comorbidities [8]. The array of available therapeutic options for DVT and the prevention of PE necessitates a nuanced understanding of each method’s benefits, risks, and context of use. In this patient, Heparin was initiated as part of the anticoagulation therapy due to the development of acute DVT following the placement of a venous catheter for plasmapheresis. The use of Heparin, particularly LMWH, is well-established in managing the acute phase of DVT, as it helps prevent further thrombus propagation and reduces the risk of PE and recurrent DVT [9]. However, heparin therapy can come with notable side effects, including bleeding [10], especially if the patient has underlying conditions that predispose them to bleeding. Additionally, patients receiving Heparin are at risk for heparin-induced thrombocytopenia (HIT) [11].

IVC filters are an established option to prevent PE in patients who have contraindications to receive anticoagulation therapy, those who experience bleeding after the initiation of anticoagulation, and those who do not show an expected increase in coagulation indices (INR, PTT) despite treatment with high-dose anticoagulation [1,2]. The insertion of the retrievable IVC filter served as a crucial intervention given the severe thrombotic event the patient faced. While effective in preventing PE, the case illustrates that these devices carry risks, such as migration and organ penetration, particularly if left in situ for prolonged periods [3,4].

Despite the intended safety this device conferred, the subsequent complications, specifically the filter struts penetrating into the abdominal Aorta, illustrate one of the significant risks associated with IVC filter placement. The majority of IVC filters are designed with expandable elements that use axial force and small hooks to prevent device migration [12]. This design may promote penetration of elements through the walls of the IVC, which increases with longer dwell times. Literature indicates that filter penetration can occur in approximately 12% of cases, often leading to life-threatening scenarios, as seen here [4]. The Society of Interventional Radiology Clinical Practice Guideline for IVC Filters recommends retrieving retrievable IVC filters as soon as protection from pulmonary embolism is no longer needed, typically within 29-54 days after placement, depending on the clinical scenario [13]. The prolonged dwell time of the filter in this patient, combined with her complex medical history, likely contributed to the adverse outcome.

## Evaluation of treatment protocols

1. Risk-Benefit Considerations: When selecting the appropriate treatment modality, the clinician must evaluate the patient’s overall clinical picture, including any contraindications to particular therapies (eg, bleeding disorders) and their comorbid conditions that may complicate management [14].
2. Monitoring and Follow-Up: Continuous assessment of patient response to therapy, including monitoring coagulation parameters in those receiving UFH, and adjusting treatment regimens based on individual responses, is vital [15]. The follow-up period is crucial for patients with IVC filters to facilitate timely removal to mitigate risks of complications such as migration and penetration [5].
3. Integration of Multidisciplinary Care: The complexity of managing patients with multiple comorbidities may necessitate a multidisciplinary approach to create comprehensive management plans tailored to individual patient profiles [14].
4. Patient Education and Involvement: Patient education on the signs and symptoms of thromboembolic events and potential complications associated with their treatments can enhance adherence to prescribed therapies and reinforce the importance of follow-up appointments.

## Future directions

This report stresses the need for further research on the long-term outcomes of IVC filter placements, particularly in highly comorbid populations. Future studies could seek to refine guidelines surrounding the use of IVC filters, emphasizing the careful selection of candidates who would truly benefit and monitoring for complications post-placement. Additionally, advancing imaging techniques could enhance the early detection of filter-related complications, facilitating timely intervention and potentially improving patient outcomes.

## Conclusion

In conclusion, this case highlights the delicate balance in managing complex patients with risks of thromboembolic events, emphasizing careful consideration of procedure risks, real-time monitoring, and the necessity for stringent follow-up protocols to mitigate complications associated with IVC filters. As the use of IVC filters continues to be a common practice in the prevention of PE, this case serves as a reminder of the potential for serious complications and the need for a personalized approach in the management of such patients.

## Ethical Approval

The Ethics Committee of Tabriz University of Medical Sciences does not require ethical approval for case reports.

## Author’s contributions

All authors read and approved the final manuscript.

## Declaration of generative AI and AI-assisted technologies in the writing process

During the preparation of this work, we used ChatGPT and EditGPT in order to improve grammar, clarity, and content quality. After using these tools, we reviewed and edited the content as needed and we take full responsibility for the content of the publication.

## Patient consent

We confirm that written informed consent has been obtained from the patient and his husband and they have approved for this information to be published in this case report.

## References

[1] Mismetti P., Laporte S., Pellerin O., Ennezat P.V., Couturaud F., Elias A. (2015). Effect of a retrievable inferior vena cava filter plus anticoagulation vs anticoagulation alone on risk of recurrent pulmonary embolism: a randomized clinical trial. Jama.

[2] Young T., Sriram K.B. (2020). Vena caval filters for the prevention of pulmonary embolism. Cochrane Database Syst Rev.

[3] Wiske C.P., Harish K., Maldonado T.S., Tapson V.F., Piazza G. (2023). PERT Consortium Handbook of Pulmonary Embolism: Research, Care, and Management.

[4] Montoya C., Rey J., Polania-Sandoval C.A., Bornak A., Shao T., Kenel-Pierre S. (2024). Inferior Vena Cava filter long term complications and retrieval techniques: a case series and literature review. Vasc Endovasc Surg.

[5] Rodriguez A.K., Goel A., Gorantla V.R. (2024). Complications associated with inferior vena cava filter retrieval: a systematic review. Cureus.

[6] Bajda J., Park A.N., Raj A., Raj R., Gorantla V.R. (2023). Inferior Vena Cava filters and complications: a systematic review. Cureus.

[7] Jia C.-y., Dai D.-d., Bi X.-y., Zhang X., Wang Y.-n. (2024). Advancements in the interventional therapy and nursing care on deep vein thrombosis in the lower extremities. Front Med.

[8] Brenner B., Hull R., Arya R., Beyer-Westendorf J., Douketis J., Elalamy I. (2019). Evaluation of unmet clinical needs in prophylaxis and treatment of venous thromboembolism in high-risk patient groups: cancer and critically ill. Thromb J..

[9] Salavati M., Arabshomali A., Nouranian S., Shariat-Madar Z. (2024). Overview of venous thromboembolism and emerging therapeutic technologies based on nanocarriers-mediated drug delivery systems. Molecules.

[10] Smith B, Khojandi A, Vasudevan R, Shafi N, Davis R. Uncovering bias in reinforcement learning for heparin treatment planning. 2023. doi:10.13140/RG.2.2.25879.24487.

[11] Cuker A., Arepally G.M., Chong B.H., Cines D.B., Greinacher A., Gruel Y. (2018). American Society of Hematology 2018 guidelines for management of venous thromboembolism: heparin-induced thrombocytopenia. Blood Adv.

[12] Anatani CM, Binkert CA, Chahin F, Chun AK, Daniele A, Gemery JM, et al. IVC filters: a review and what’s new. Society of Interventional Radiology; 2009. Available at: https://divcomrad.com/wp-content/uploads/2013/08/Filters-SIR-2009.pdf. Accessed October 2, 2022.

[13] Kaufman J.A., Barnes G.D., Chaer R.A., Cuschieri J., Eberhardt R.T., Johnson M.S. (2020). Society of Interventional Radiology Clinical Practice guideline for inferior Vena Cava filters in the treatment of patients with venous thromboembolic disease: developed in collaboration with the American College of Cardiology, American College of Chest Physicians, American College of Surgeons Committee on Trauma, American Heart Association, Society for Vascular Surgery, and Society for Vascular Medicine. J Vasc Interv Radiol.

[14] Muth C., Glasziou P.P. (2015). Guideline recommended treatments in complex patients with multimorbidity. BMJ: Br Med J.

[15] Samimi M.N., Hale A., Schults J., Fischer A., Roberts J.A., Dhanani J. (2024). Clinical guidance for unfractionated heparin dosing and monitoring in critically ill patients. Expert Opin Pharmacother.

